# Exploring the Relationship between Ecosystem Services under Different Socio-Economic Driving Degrees

**DOI:** 10.3390/ijerph192316105

**Published:** 2022-12-01

**Authors:** Tiantian Ma, Qingbai Hu, Changle Wang, Jungang Lv, Changhong Mi, Rongguang Shi, Xiaoli Wang, Yanying Yang, Wenhao Wu

**Affiliations:** 1 Agro-Environmental Protection Institute, Ministry of Agriculture and Rural Affairs, Tianjin 300170, China; 2 Tianjin Key Laboratory of Hazardous Waste Safety Disposal and Recycling Technology, School of Environmental Science and Safety Engineering, Tianjin University of Technology, Tianjin 300384, China; 3 Procuratoral Technology and Information Research Center, Supreme People’s Procuratorate, Beijing 100041, China; 4 Key Laboratory Environment Factors Control Agriproduct Qual Safe, Ministry of Agriculture and Rural Affairs, Tianjin 300170, China

**Keywords:** ecosystem services, InVEST model, classified management, grey correlation degree, correlation analysis

## Abstract

The large-scale transformation of natural ecosystems to socio-economic development land types under human activities was a primary reason for the decline of regional ecosystem services. It is a key issue for regional ecosystem planning and management to reveal the relationship between ecosystem services of different land use types under different socio-economic driving degrees. However, the current related research was not in-depth. Based on the land use data of Wuhan City in 1980, 1990, 2000, 2010, and 2020, this study classified land use into three categories according to the different degrees of human activities on natural ecosystem development: the land use of a natural ecosystem (LUNE), the land use of a productive ecosystem (LUPE), and the land use of a socio-economic system (LUSE). The InVEST model was used to simulate five ecosystem services (grain yield, water yield, carbon storage, habitat quality, and water purification), and the spatio-temporal distribution and functional transformation of the three land use types were analyzed. Results showed that with the intensified urban expansion in Wuhan, the LUSE types increased to 2.7 times that of the original. However, the natural land types basically maintained a stable area, coupling with the large-scale transformation between the LUPE and LUSE types. Land use change resulted in significant spatial changes of five ecosystem services, especially carbon storage and habitat quality. The correlation analysis indicated that the five kinds of ecosystem services mainly showed a synergistic relationship, meanwhile the LUSE type denoted the most significant correlation with ecosystem services among these three category types. This study indicated that besides the protection of natural ecosystems, the LUSE type would become the key land use type in the planning and management of improving regional ecological function.

## 1. Introduction

The continuous human reclamation of nature has formed a variety of land use and land cover (LULC) types [1]. Different socio-economic behaviors and different degrees changed the original structures and functions of the natural environment [2]. Pure natural protectionism or human development was of relatively little significance [3]. The balance between natural protection and human development has always been key in regional ecosystem planning and management [4]. Therefore, through changes among LULC and its functions that reveal the relationship between the social and natural driving forces on a regional scale, the research would provide significant scientific guidance for subsequent ecological environment planning and management.

Ecosystem services are the benefits that humans continuously receive from nature [5], which include tangible products or intangible services, which in turn improve human well-being and contribute to social development [6]. Human survival and development are inseparable from ecosystems [7]. The rapid growth of population and the vigorous development of the economy has caused the deterioration of the ecological environment [8]. The broken ecological balance was not conducive to the sustainable development of the ecosystem [9]. However, with the improvement of ecological protection awareness, woodlands, wetlands, and other natural ecosystems have been vigorously protected and restored. Exploring the ecosystem service function and developing the ecosystem appropriately and reasonably are the basics for the harmonious coexistence between humans and nature [10]. At present, at the level of regional LULC pattern, studies analyzing the changes of various ecosystem services of various land use types dominated the mainstream [11]. The driving force analysis after the dynamics of ecosystem services and land use types was mainly related to statistical data such as regional economic development and population growth, which, in fact, isolated the direct relationship between the driving force, LULC, and ecosystem services [12]. LULC is used as a link to explore the changes in various ecosystem services driven by human or nature, and to reveal the key factors affecting regional ecological functions in the interaction between human activities and nature conservation, thus becoming an innovative study to guide regional ecological planning and management.

Due to the introduction of ecosystem services, domestic and foreign scholars have conducted in-depth research on the connotation, classification, evaluation methods, and value estimation of ecosystem services [13,14]. In recent years, with the maturity of geographic remote sensing, artificial intelligence, and GIS technology, more than 10 kinds of ecosystem service function assessment models have been generated, such as InVEST (the Integrated Valuation of Ecosystem Services and Trade-offs) model, ARIES (Artificial Intelligence For Ecosystem Services) model, and EPM model (Ecosystem Portfolio Model) [15]. The InVEST model developed by Stanford University, the Nature Conservancy (TNC), and the World Wide Fund for Nature have been widely used. The model was a distributed algorithm based on 3S technology, which could visualize and quantitatively evaluate 13 sub-modules such as water yield, soil erosion, water quality purification, and habitat risk in action space [16]. Choudhary used the Habitat Quality module of InVEST model to analyze the impact of the increase in land cover and mesquite on the Habitat Quality of wetland parks in different contexts in India, so as to provide technical support for solving the problems of species invasion and water shortage [17]. Bao Yubin et al. evaluated the habitat suitability of wetland in Yellow River Nature Reserve through the habitat quality module of InVEST model [18], and indicated that through comparison with other land use types, wetland has the highest suitability and was more suitable for amphibians and fish. Wang Meng and Guo Yun, respectively, explored the synergies and trade-offs between ecosystem services under different land use situations in Tianjin and Dongting Lake using InVEST model, which provided a theoretical basis for promoting sustainable ecosystem development [19,20]. Wei Peijie analyzed the annual water output of the upper reaches of the Shule River over the past 20 years at the watershed scale, providing scientific reference for regional water resource protection planning [21]. Zhang explored the impact of urban expansion by analyzing the hot-spot spatio-temporal dynamics and trade-offs and coordination of ecosystem services [22].

It could be seen that the studies on ecosystem services simulations based on InVEST have provided extensive support for ecosystem planning and management around the world. However, most researchers’ studies on ecosystems have focused on natural ecosystems, such as wetlands, woodland and grassland, or ecological functions such as biodiversity, water purification, and water conservation based on them. Therefore, current ecosystem management strategies focus on the protection of natural ecosystems [23]. According to the degree of the exploition of human activities on natural ecological systems, land use can be divided into three categories: the natural ecosystem types, the keeping some ecological function and producing value as part of a productive ecosystem, and the social and economic land use types. The interactive characteristics of the three types of land use in ecosystem services could reflect the law of regional ecosystem development driven by human activities and provide a scientific basis for regional ecological planning and management of comprehensive social and economic development.

The multiple LULC types could be divided into different categories according to different rules. In order to better deal with the relationship between nature conversation and socio-economic development in the planning and management of ecosystems, exploration of the relationship between socio-economic and natural drivers has been suggested. This research took Wuhan as the case study area, which was a typical first-tier urban city with a large population base and rapid economic development. The dynamics of multiple LULC types and ecosystem services were also obvious in Wuhan [24,25,26]. Based on the InVEST model and LULC data of Wuhan in 1980, 1990, 2000, 2010, and 2020, this paper tried to explore and reveal the trajectory of land use transformation of three LULC categories, which represent the typical land use types driven by natural or socio-economic factors. The temporal and spatial changes of five ecosystem services for three LULC categories, such as grain yield, water yield, water purification, carbon sequestration, and habitat quality, were modeled and analyzed qualitatively. Further, the correlations among the five ecosystem services for the three LULC categories were explored. Suggestions were proposed as the reference for the correct treatment of natural and economic ecosystem construction.

## 2. Materials and Methods

### 2.1. Study Area

**Figure 1:**
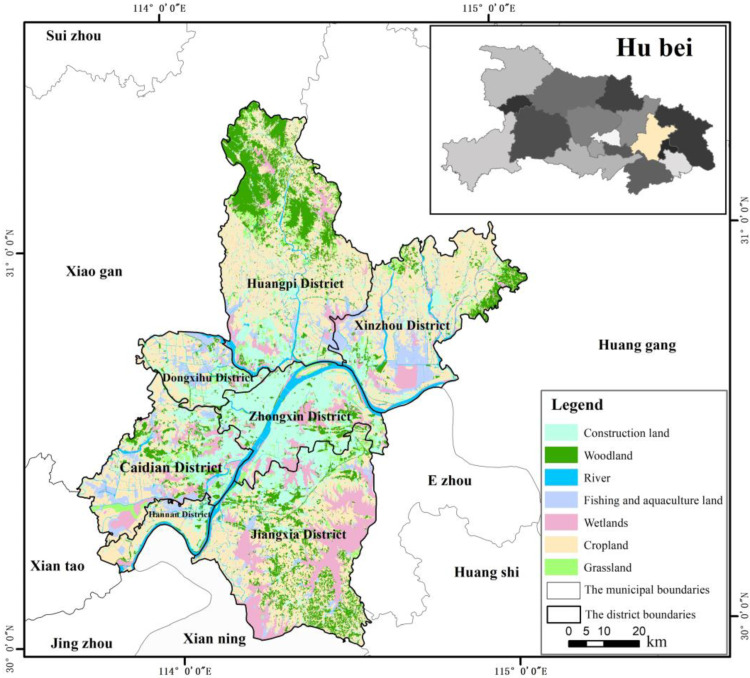
The location of study area.

Wuhan City was selected as the case study area (Figure 1) with particularly prominent contradiction between economic development and nature protection caused by urbanization. It was located in the subtropical monsoon climate zone, with abundant rainfall throughout the year. It consisted of 7 central urban areas and 6 outer suburbs, with a total area of 8569 km^2^, of which, water area accounts for 24.7%. It has a permanent resident population of 12.45 million and a GDP of 965.64 billion yuan. It was called “a first-tier city floating on water”. Under the background of accelerated urbanization, the population size has increased rapidly, and the urbanization construction has a significant impact on the change in land use types in Wuhan, affecting the economic production structure and the natural ecological environment.

### 2.2. Classification of Land Use and Land Cover

The land use data of Wuhan City in 1980, 1990, 2000, 2010, and 2020 were employed to explore the overall changes of LULC and ecosystem services in the past 40 years and were generated by visual interpretation of remote sensing images and provided from Beijing Normal University. The data source of 1980 was an MSS (multispectral scanner) image, and the other four years were derived from Landsat remote sensing images with 30 m × 30 m accuracy. As shown in Table 1, referring to the Chinese National Standard “Classification of Land Use Status” (GB/T 21010-2017) and the characteristics of LULC, the 18 secondary categories of the original LULC data were fused into 7 primary categories: cultivated land, forest, grass, wetlands, river channel, aquaculture and fishery, and construction). In addition, according to the different degrees of human activities on natural ecosystem development coupling with the different functions of land use types in ecosystem services, the seven land use types were classified according to three categories: the land use of natural ecosystem (LUNE), the land use of productive ecosystem (LUPE) as mainly supporting the economic functions, and the land use of socio-economic system (LUSE). The LUNE mainly supported the ecological functions such as the carbon sequestration, biodiversity protection, and water purification. The LUPE mainly generated economic production functions, but also supported a few ecological functions. However, the LUSE represented the land types directly related to social and economic development without ecological functions.

### 2.3. Simulation of Ecosystem Services

Five ecosystem services were simulated by sub-modules using the InVEST model. Detailed operating principles of these five sub-modules can be found on the official website of InVEST model (https://naturalcapitalproject.stanford.edu/software/invest, accessed on 5 March 2022).

#### 2.3.1. Grain Production

The Crop Production Percentile module of InVEST was applied to simulate the rice yield of Wuhan. The InVEST Crop Production Percentile model produced estimates of 175 crops’ yield from existing data, percentile summaries, and observed yields. Operating model data needed a directory to model data, land-use/land-cover map, and landcover to crop table. Land use/land cover (column lucode) must be mapped to integer a code name of crops (column name crop_name). According to the statistical yearbook of Wuhan in 2021, the rice was the main grain crop planted, and the yield of rice accounted for 86% of the total grain output of Wuhan. Therefore, in this simulation the rice was one of the accepted 175 crops for the percentile model.

#### 2.3.2. Water Yield

Based on the principle of water balance, the water yield module of InVEST combined parameters such as climate, topography, hydrology, LULC, and soil properties to calculate water yield. The amount of water produced within each grid cell was reflected by the difference between the input and output water, that is, the amount of water produced within each grid cell was equal to the amount of precipitation minus the actual evapotranspiration. In this study, as shown in Table 2, all parameters and data required for model operation in this study were from official and published reliable data and processed in accordance with the requirements of model operation.

#### 2.3.3. Carbon Sequestration

Ecosystems can store carbon in the biomass of plants, soil, and other components, and adjust to climate change by absorbing and fixing free carbon in the atmosphere [27]. On a regional scale, LULC change was the main driving force of carbon storage change [28]. In the carbon sequestration module of InVEST, the total carbon sequestration of a land use type was approximately the sum of the carbon storage of aboveground organisms (all living plants), underground organisms (plant roots), soil carbon storage (mineral and organic soil), and dead organic matter [29]. Secondary LULC classification data were used in the operation of this module, and the carbon densities of aboveground carbon, underground carbon, soil organic carbon, and dead organic carbon of each land use type were all referred to the relevant literature and investigations [30,31,32] (Table 3).

#### 2.3.4. Nutrient Delivery Ratio

Purification capacity was the key factor to evaluate water quality. The transformation of LULC caused the destruction of natural vegetation, resulting in soil and water loss. The interception and adsorption ability of vegetation and soil to N, P, and other nutrients were weakened. The decreased water quality caused the balance of the ecosystem. The nutriment delivery ratio (NDR) module of InVEST determined the nutriment quantity of each LULC type based on the nutriment load, the nutriment efficiency, the nutriment distance of the nutriment quantity, and the proportion of dissolved nutriments to the nutriment quantity. The required rainfall data and soil data were referred to the water yield module, and the biophysical attribute table were collected from the existing study [33,34,35].

#### 2.3.5. Habitat Quality

The habitat quality of Wuhan City was simulated based on the Habitat Quality module of InVEST. In the model, LULC types that affect or destroy natural habitats such as wetlands and woodland were considered as threat sources, and the impacts of human activities and other threat factors on biodiversity of different land use types were comprehensively evaluated by simulating the impacts of threat sources on natural land use types [36]. The relative index of habitat quality and the index of habitat degradation degree were calculated by the data of land use type information, threat sources, and habitat response to threats.

As the significant land use types of human activities, five land use types including the paddy field, dry land, rural residential areas, towns, and other construction land were set as the threat factors. The threat source distance and weight, threat space attenuation type, and the sensitivity of the threat factors of different land use types were determined through the relevant studies (Table 4) [37,38].

### 2.4. Correlation and Correlation Analysis

Correlation analysis analyzed two or more variable elements with correlation to measure the degree of correlation between variable factors. In this study, the Pearson correlation coefficient method in SPSS software was used to analyze the correlation of five ecosystem services, and the synergistic and trade-off relationship between each two services were quantitatively explored. The calculation formula was as follows:

(1) $$r=\frac{1}{n-1}\sum_{i=1}^{n}\left(\frac{X_{i}-\bar{X}}{s_{X}}\right)\left(\frac{Y_{i}-\bar{Y}}{s_{Y}}\right)$$

## 3. Results

### 3.1. The Dynamics of LULC Types

**Figure 2:**
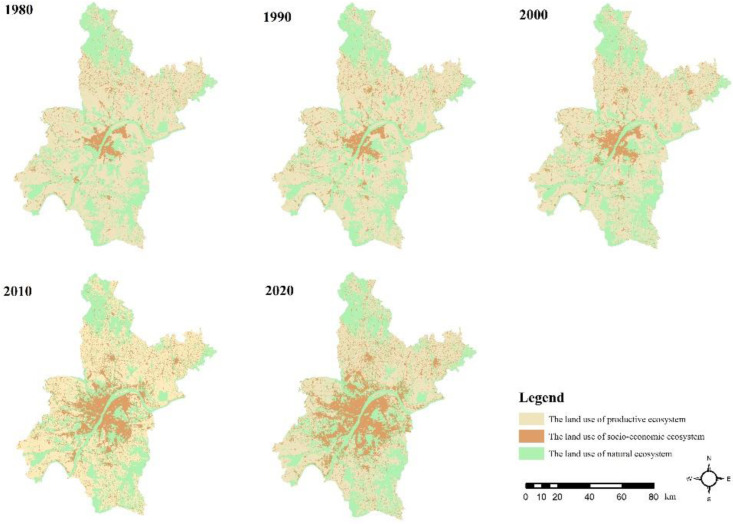
The pattern of three LULC categories in Wuhan city during 1980–2020.

As shown in the Figure 2, the area of the LUSE type was the largest type among the three classified types during the forty years from 1980s to 2020s, following the LUPE and LUNE types. At the same time, the area of the LUPE and LUSE, respectively, showed a decrease and increase, and the area of LUNE tended to be stable (Figure 2). In these 40 years, the LUSE type kept going down occupying from the 61.63% to the 44.77%, whereas the LUPE type sustained an increased from a 7.99% occupation rate to a 21.64% one. The proportion of the LUNE type of area has always been about 30%.

**Figure 3:**
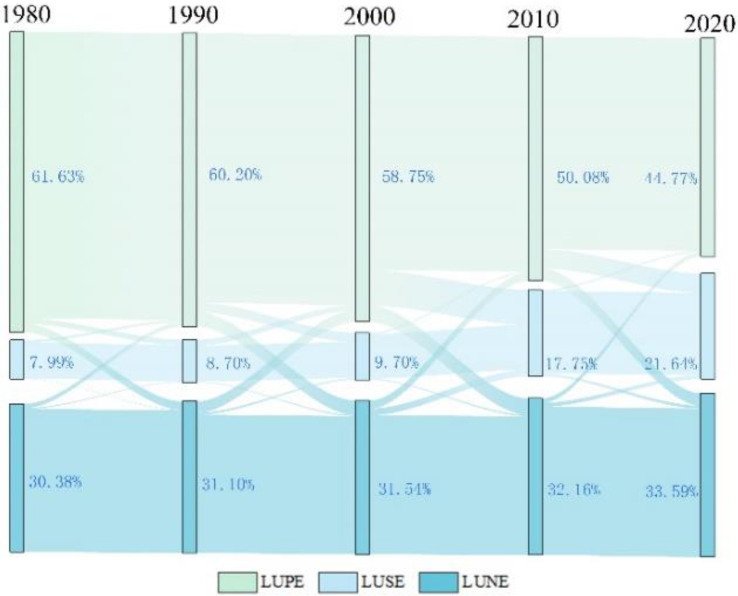
The conversion among three LULC categories in 1980–2020.

From 1980 to 2000, the LULC change was relatively stable and the area of each LULC type remained stable. After 2000, influenced by urban expansion and rapid economic development, the LUSE types increased significantly, whereas the LUPE and LUNE types gradually decreased. However, the LULC conversion mainly occurred between the LUPE and LUSE types. The area of three classified LULC types coupled with LULC types dramatically changed during the 2000 to 2020. In this period, as the strongest conversion, the total transfer area reached 618 km^2^, which accounted for 7.2% of the total area of Wuhan. Following from 2010 to 2020, the 336 km^2^ of the area of LULC types were transferred, accounting for 4% of the total area of Wuhan (Figure 3).

### 3.2. The Changes in Ecosystem Services

**Figure 4:**
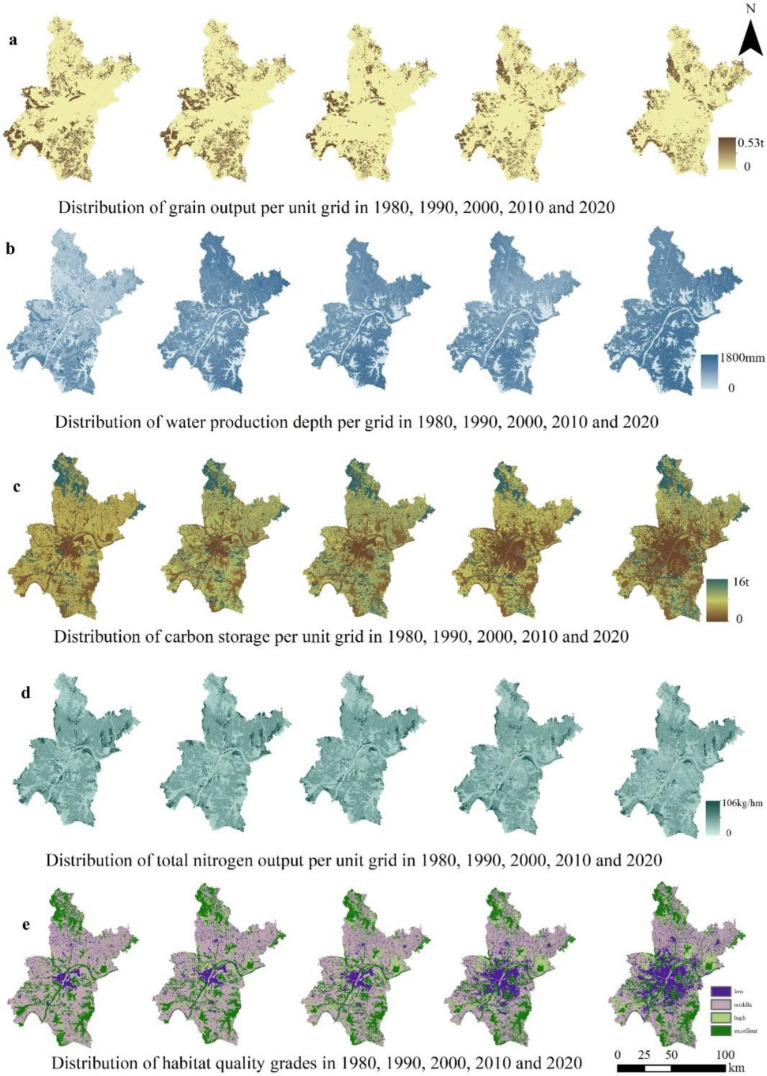
The conversion among three LULC categories in 1980–2020.

**Figure 5:**
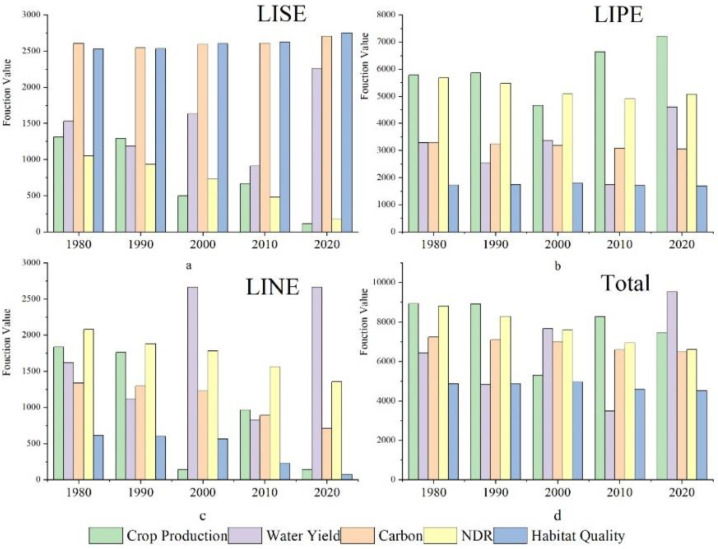
The dynamics of five ecosystem services for three LULC categories in 1980–2020.

The results showed that there were significant differences in variation among the five ecosystem services (grain yield, water yield, water quality purification, habitat quality, and carbon storage) in Wuhan during 1980–2020 (Figure 4 and Figure 5). Influenced by climate conditions and rapid economic development, the ecosystem services of grain yield, habitat quality, and carbon sequestration function all showed a downward trend. The water purification and water yield increased.

The statistic units of crop production, water yield, carbon storage and nutrient delivery were million tons, $10^{6}$ mm, $10^{4}\;$ Mg and ton, respectively.

#### 3.2.1. Crop Production

The grain yields in 1980, 1990, 2000, 2010, and 2020 were 8.97 × 10^5^ t, 8.95 × 10^5^ t, 5.93 × 10^5^ t, 8.32 × 10^5^ t, and 7.53 × 10^5^ t, respectively. A U-shaped change that decreased and then increased. The grain yield in 2000 was the lowest. Compared with 1990, the grain yield in 2000 decreased by 33.74%. The spatial difference of grain supply was subtle. The areas with high rice yield were mainly concentrated in Jiangxia District, the east of Caidian District, the southeast of Dongxihu District, and the north of Xinzhou District. After 2010, the rice yield in the east of Huangpi District increased, and the rice yield in the central urban area was the least (Figure 5).

#### 3.2.2. Water Yield

The average water depth of Wuhan decreased from 1084.8 mm in 1980 to 955.5 mm, and greatly increased to 1368.05 mm in 2000. The maximum water depth of Wuhan in five periods fluctuated from 809.525 mm to 1744.34 mm, among which the water depth of Wuhan in 2010 was the lowest. In 2020, the water production depth of the fifth stage showed a “W” shape trend of declining, rising, declining, and rising again. The water production depth of the fifth stage showed a consistent pattern in space, with high water production depth in the north and low water production depth in the south (Figure 5).

#### 3.2.3. Carbon Sequestration

Generally, the carbon sequestration in the five periods showed a decreasing trend, and an increasing trend from the center to the periphery in space. The total amount of carbon sequestration in the northern natural terrane was the largest. The carbon storage in 1980 and 2020 was 7.26 × 10^7^ t and 6.49 × 10^7^ t, respectively, with the total decreasing carbon sequestration of 0.77 × 10^7^ t and the rate of 10.6%. From 2000 to 2010, the carbon storage declined the most, reaching 0.43 × 10^7^ t, whereas other periods showed a trend of decreasing by about 1 × 10^6^ t every decade.

Comparing the changes of carbon sequestration of the three classified LULC types, the LUSE type has had the most obvious change in the past 40 years and has increased nearly three times. The total carbon of the LUPE decreased from 45.36 × 10^6^ t to 30.76 × 10^6^ t, which is a decrease of 15%. The total carbon of the LUNE increased slightly. The carbon density of LUNE tended to be stable. The carbon density of the LUNE and LUPE type decreased by 1.0 kg/hm and 5.7 kg/hm, respectively, whereas the LUSE type increased by 2.7 kg/hm.

#### 3.2.4. Nutrient Delivery Ratio

The simulation results showed that the spatial difference of nitrogen output load was small, and the high value areas were mainly distributed in the peri-urban and productive land types. Over the past forty years, the total output of nitrogen in Wuhan has decreased by 2198 tons, which was a continuous decline. The average nitrogen output load decreased from 10.48 kg/hm in 1980 to 7.88 kg/hm in 2020. The average nitrogen load decreased from 9.87 kg/hm to 9.04 kg/hm during 1990 to 2000. From 2010 to 2020, the nitrogen load changed the least, and only decreased by 0.4 kg/hm.

#### 3.2.5. Habitat Quality

There was a certain spatial consistency in the change trend of habitat quality in 1980, 1990, 2000, 2010, and 2020, and the overall spatial distribution of habitat quality was also similar. According to the equal interval method, the habitat quality scores were divided into four categories from poor to good: low, middle, high, and excellent. The low grade of the habitat index was mainly distributed in the central urban area of Wuhan, whereas the north, southeast, and northeast had an excellent grade of habitat throughout all the periods (see Figure 4). From the perspective of temporal dynamics, the habitat grade mainly developed from low to middle, and the average of habitat index in the five periods, respectively, were 0.4339, 0.4320, 0.4360, 0.4258, and 0.4280. The overall habitat suitability in Wuhan decreased, but the average of habitat index in 2010 was the highest. The grade of low habitat increased from 8% of the total area in 1980 to 22% in 2020, mainly transferred from low and middle grades. The area of excellent grade did not change obviously and remained between 24% and 25% of the total area in all periods.

### 3.3. The Correlation of Ecosystem Services

#### 3.3.1. Ecosystem Service Function Coordination and Balance

**Figure 6:**
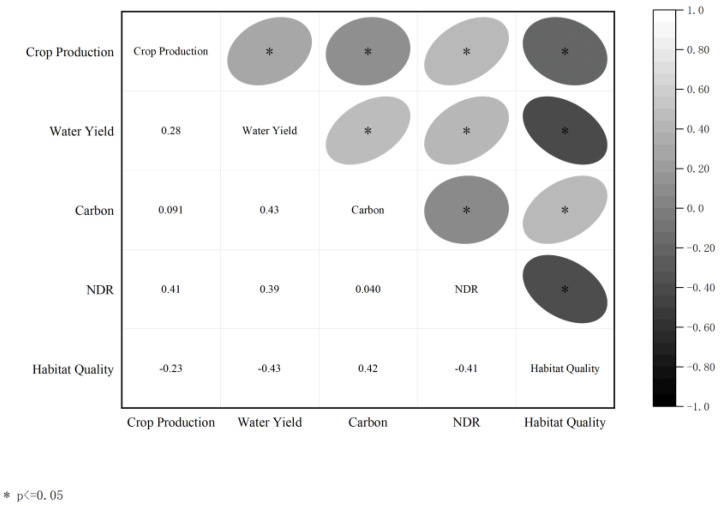
The correlation of five ecosystem services.

As shown in Figure 6, among the five ecosystem services, pairwise different services were combined into 20 groups of relationships, and all showed significant correlation. Habitat quality was negatively correlated with grain yield, water yield, and water purification, which indicated a trade-off relationship, whereas other pairwise services were positively correlated with each other, denoting a synergistic relationship. The synergy between carbon storage and water yield was the highest (0.429), and the synergy between carbon storage and grain yield was the lowest (0.091). The most significant trade-off was between habitat quality and water yield (−0.429), and the least trade-off was between habitat quality and grain yield (−0.231).

#### 3.3.2. Synergy and Tradeoffs between Different LULC Classes and Ecosystem Services

**Figure 7:**
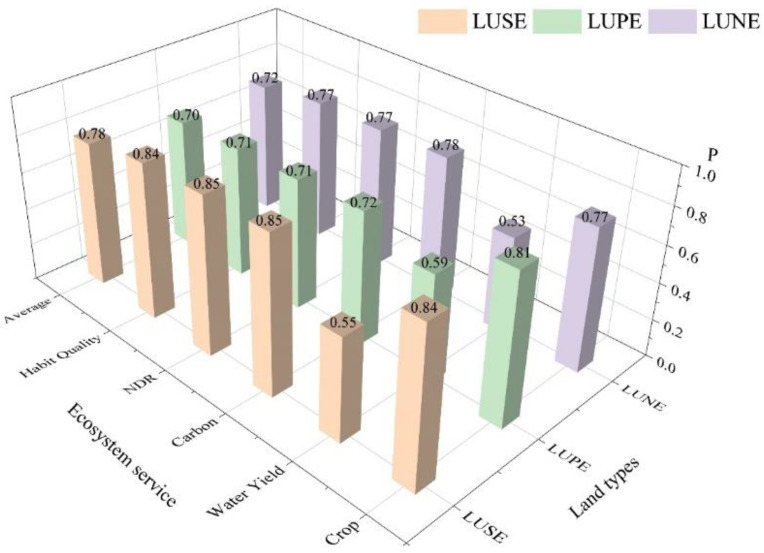
The correlation between LULC classes and ecosystem services. The total ecosystem services were calculated by the average of five ecosystem services.

Grey correlation analysis was used to quantitatively discuss the relationship between LULC types and five ecosystem services. The correlation degree between three LULC classes and five ecosystem services were from 0.53 to 0.85, with an average correlation degree of 0.73. The results showed that LULC type had a great influence on ecosystem services in Wuhan in 1980–2020. (Figure 7) Generally, the correlation degree between the LUSE types and grain yield, carbon storage, water purification, and habitat quality were strong, and all the gray correlation degrees were above 0.8, indicating that the changes of social-economic land types were closely related to the four ecosystem services. The LUPE and the LUNE types most impacted the grain production and carbon storage with the higher correlation degree, respectively. However, all the correlation degree of water production with the LUNE, LUPE, and LUSE were less than 0.6.

## 4. Discussion

### Analysis of Ecosystem Services

In the past 40 years, the socioeconomic types of land in Wuhan showed a rapid growth trend, and the service capacity of product supply, water purification, and climate regulation of Wuhan’s ecosystems declined. The spatial variation of service function was mainly affected by the spatial distribution of three types of land use.

Grain output was affected by agricultural production conditions, natural disasters, science, and technology [39]. When the level of agricultural production was relatively stable, the direct reason for the decrease in grain yield was the gradual decrease in planting area. In 1998, the great flood in the Yangtze River Basin caused serious inundation of paddy fields and the loss of farmland, which was difficult to recover for a short time, leading to a sharp decline in grain output in Wuhan in 2000. In order to ensure food security, the state abolished agricultural tax in 2006, promulgated the policy of encouraging farmers to plant fields and grains, and put forward the permanent basic farmland in 2008, which ensured the planting area of grain. Coupled with scientific and technological progress and scientific planting, the grain output was restored to 8.32 × 10^5^ t by 2010. Although the productive land area has been greatly reduced in the past 40 years, the grain yield has not been reduced due to advanced science and technology such as hybrid rice, suggesting that human intervention has become an important dominant factor.

Water yield function was closely connected with water resource conservation, and the simulation results provided an important reference value for the sustainable development of water resources in Wuhan. The amount of water yield was mainly related to precipitation [40], but also affected by physical geography and social economy [41]. The precipitation in the southern part of Wuhan was greater than that in the northern part, so the higher value of water depth was mainly concentrated in the southern part. In terms of land types, natural types such as woodland, grassland, and wetland has a high coverage rate of surface vegetation and a large potential evapotranspiration, resulting in a low depth of water production. However, the economic land has less vegetation coverage and more impermeable area, and the precipitation penetration into the underground and potential evapotranspiration were less, so the water yield was relatively more.

N, P, and other nutrients were the main components causing the deterioration of water quality. The simulation results of total nitrogen denoted that the high value area of nitrogen output load mainly existed in economical and productive types of soils. The total nitrogen output varies in space, and the level of water purification service function was affected by topography, land use types, and other aspects [42]. The elevation difference between the north and south of Wuhan was large. In the north, the terrain was high, mainly low hills and hills, whereas in the middle and south, the terrain was flat, mainly plain, with a maximum elevation difference of 854 m. In the high terrain, the runoff flow rate of rainfall was relatively fast, and the rejection rate of N, P, and other nutrients on the surface was low, so the total nitrogen output in the northern region was higher than that in the southern region. In contrast, the northernmost and southeastern areas of Wuhan were mainly natural types with dense forests, which have obvious retention and adsorption effects on nitrogen, phosphorus, and other nutrients, which were the main reasons affecting the effect of water purification.

The rise of factories and residential living areas in economic land category led to the increase in domestic pollution discharge of industrial wastewater and the weakening of water purification capacity [26]. Due to the massive release of pesticides and fertilizers, which exceeded the absorption of the soil and planted crops, residual nutrients such as N and P were retained in soil moisture, which was the main reason that the productive land became the area with a high nitrogen element load [43]. In order to ensure the safety of water resources and improve the water ecological environment, the government issued a series of policies, such as the Wuhan Water Pollution Prevention Plan and the Wuhan Water Quality Improvement Action Plan, to improve the water quality purification capacity and protect the water ecological environment in Wuhan.

From 2000 to 2010, carbon storage declined the most. Due to the acceleration of economic construction and the continuous progress of urban construction, urban expansion led to the transfer of productive and natural land types with high carbon density to economic and social land types with low carbon density [44], which was the root cause for the biggest decline of carbon storage in these ten years. However, with the promulgation of the policy of returning farmland to forest or grassland, the land with a strong carbon sequestration capacity, such as woodland and grassland, has increased in recent years.

Habitat quality directly reflected the maintenance of biodiversity and ecosystem health [45]. An analysis of habitat grade evaluation results showed that more and more areas with low habitat grade were concentrated in the central part of Wuhan over time, mainly due to the accelerated expansion of the central urban area. As a result, the productive land and natural land with good or excellent habitat suitability were transferred to economic land, increasing the urban construction area, and intensifying human activities. The ecological balance was broken, which resulted in more and more areas with poor habitat grade and accelerated the degradation of habitat. On the contrary, the southernmost and northernmost areas of Wuhan were mainly natural types with low intensity of human activities and mostly biological favorable vegetation such as forests and grasses, which created favorable living conditions for the survival of biodiversity and have been in the excellent class for the past four decades. The existing area with a high habitat grade was suggested to be classified as the priority protection area in planning. At the same time, it was also suggested to reassess the suitability of biodiversity in low habitat grade areas and carry out targeted ecological restoration.

No ecosystem service existed in isolation, and the coordination and trade-off of ecosystem services is a hot topic for scholars nowadays [25,46]. The results showed that the interrelationship among five ecosystem services in Wuhan were mainly synergistic, denoting they were mutually promoted. The trade-offs relationship only behaved between habitat quality and grain yield, habitat quality and water yield, habitat quality and water quality purification, indicating the growth of food crops needs the nourishment of chemical fertilizers, and the use of chemical fertilizers was not conducive to the livability of insects and other organisms. Places with high water yield and high output of nutrients such as nitrogen and phosphorus were also not suitable for the survival of organisms.

Recently, in China, the protection of the ecological environment has gained unprecedented attention [47]. At present, the country has a perfect natural ecosystem protection system, which mainly focuses on protecting natural areas [48]. However, productive ecosystems such as croplands and aquaculture ponds, types that mainly promoted social and economic production but still retained natural ecological functions, are neglected when it comes to conservation in cooperation with natural ecosystems. The present study indicated that the area increases in LUSE type on behalf of urban construction expansion mainly occupied the LUNE type representing natural ecosystems before 2000. However, since 2000, a series of measures and policies in natural ecosystem production were conducted, such as the natural wetlands or forest parks, nature protected area, and biodiversity production [49]. Therefore, after 2000, the study showed the big area of the LUPE denoting the productive ecosystem type were transferred to the LUSE. In the expansion of large-scale urban development, Wuhan adopted the natural ecosystem protection policy at the same time, so the rapid urbanization had relatively little impact on the area and function of the natural ecosystem. However, at the same time, a large area of productive ecosystem was occupied, which undermined the ecosystem services that productive ecosystems still maintain. The overall ecosystem services decreased in Wuhan, indicating that the protection of natural ecosystems was insufficient to compensate for the overall reduction in regional ecosystem services caused by the large-scale occupation of productive ecosystems. Therefore, in order to maintain and strengthen the protection of natural ecological system, it is suggested that the regional construction of ecological environment in Wuhan planning should be coordinated urban development and agricultural development or fisheries aquaculture. It is suggested that by the optimization development pattern, considering the relationships among different LULC and ecosystem services at a regional scale, overall regional ecosystem services can be improved.

## 5. Conclusions

To explore the relationship among the land use of a natural ecosystem (LUNE), the land use of a productive ecosystem (LUPE), and the land use of a socio-economic system (LUSE), this study analyzed the transformation of LULC and ecosystem services for these three LULC-classified types based on the LULC data for Wuhan City in 1980, 1990, 2000, 2010, and 2020. The LULC changes mainly focus on the transfer between LUPE and LUSE. Influenced by the urban expansion of economic development, the LUSE type expanded by 2.7 times, with the LUPE area decreasing by nearly 30%. The area of LUNE type remained stable. During the past 40 years, the ecosystem services in Wuhan decreased significantly, including a 10.6% decrease in carbon storage, a 12.4% increase in poor habitat area, a 16% decrease in grain yield, a 33% decrease in total nitrogen output per unit area, and a 32% increase in total water yield. Generally, the five ecosystem services showed synergistic with each other, and had the highest correlation with the economic type, which showed that they were most affected by the economic type. Coupled with the protection policy of natural ecosystem, the LUPE type played the key role in regional ecological environment.

## Figures and Tables

**Table 1:** The classification of land use and land cover.

Classification	The Primary Classification	Reclassify
The land use of productive ecosystem (LUPE)	Cropland	Paddy field
		Dry land
	Fishery land	Fishery land
The land use of natural ecosystem (LUNE)	Forestland	forestland
		Shrubland
		open woodland
		other woodland
	Grassland	Grassland with high coverage
		Grassland with moderate coverage
		Grassland with low coverage
	Wetlands	Lakes
		Reservoir
		Swag
		Graff
The land use of socio-economic ecosystem (LUSE)	Construction land	Urban land
		Village land
		Other construction land

**Table 2:** Data sources of water production.

Data	Method	Source
Administrative boundaries	Using Arcgis for tailoring	Resources and Environment Science and Data Center offers Chinese municipal and county-level administrative boundary data
Average annual precipitation	Kriging method	National meteorological science data center of Wuhan city and the surrounding 16 weather stations from 1980 to 2020 the day precipitation, average temperature, and daily maximum and minimum temperature data
Soil	Using Arcgis for extraction	Chinese soil data set based on world soil database (hwsd) (v1.1) This data is from: National Cryosphere Desert Data Center
Average annual reference evapotranspiration	Modified—Hargreaves formula	Extraterrestrial top radiation from the sun radiation value data from FAO # 56 irrigation water file attachment 2
Plant available water content	SPAW software to calculate	National Cryosphere Desert Data Center
Z parameter	Manual debugging	Water resources in Wuhan city gazette in 2016–2020

**Table 3:** Carbon density of Land use/land covers.

Serial Number	LULC (Mg/hm^2^)	Carbon Stored in Aboveground Biomass (Mg/hm^2^)	Carbon Stored in Belowground Biomass (Mg/hm^2^)	Carbon Stored in Soil (Mg/hm^2^)	Carbon Stored in Dead Organic Matter (Mg/hm^2^)
11	Paddy field	6.17	6.48	76.35	1.07
12	Dry land	6.19	6.28	68.26	1.07
21	Forestland	23.73	32.1	93.81	18.12
22	Shrubland	19.5	8.23	59.66	1.87
23	Open woodland	22.04	19.05	88.37	9.37
24	Other woodland	22.14	8.93	85.2	2.82
31	Grassland with high coverage	6.82	8.78	52.83	4
32	Grassland with moderate coverage	6.98	8.78	52.83	3.2
33	Grassland with low coverage	6.28	8.78	63.39	5
41	Graff	1.88	4.14	34.5	3.98
51	Urban land	3.21	2.98	28.91	0
52	Village land	1.61	2.98	29.16	0
53	Other construction land	3.21	2.98	29.41	0
401	Reservoir	1.88	4.14	36.17	3.98
402	Lakes	1.88	4.14	36.5	3.98
403	Swag	2.79	0.37	36.83	3.98
404	Fishery	2.79	0.37	37.17	3.98

**Table 4:** Habitat suitability and habitat sensitivity parameters to threat factors.

Serial Number	LULC	Habitat	Paddy Field	Dry Land	Rural Residential Areas	Construction Land	Urban Land
11	Paddy field	0.4	0	1	0.35	0.1	0.5
12	Dry land	0.3	1	0	0.7	0.1	0.5
21	Forestland	1	1	1	0.7	0.6	0.9
22	Shrubland	0.7	0.7	0.9	0.4	0.2	0.6
23	Open woodland	0.6	0.8	0.5	0.6	0.65	0.8
24	Other woodland	0.4	0.8	0.5	0.6	0.7	0.8
31	Grassland with high coverage	0.65	0.8	0.8	0.5	0.25	0.8
32	Grassland with moderate coverage	0.55	0.8	0.8	0.55	0.3	0.7
33	Grassland with low coverage	0.35	0.8	0.8	0.65	0.35	0.75
41	Graff	1	0.3	0.2	0.6	0.5	0.8
51	Urban land	0	0	0	0	0	0
52	Village land	0	0	0	0	0	0
53	Other construction land	0	0	0	0	0	0
401	Reservoir	1	0.9	0.2	0.7	0.5	0.8
402	Lakes	0.6	0.7	0.2	0.5	0.1	0.3
403	Swag	0.5	0.2	0.2	0.5	0.1	0.3
404	Fishery	0.6	0.3	0.2	0.5	0.1	0.3

## Data Availability

Not applicable.

## References

[1] Ma T., Li X., Bai J., Cui B. (2019). Tracking three decades of land use and land cover transformation trajectories in China’s large river deltas. Land Degrad. Dev..

[2] Wong B.B.M., Candolin U. (2014). Behavioral responses to changing environments. Behav. Ecol..

[3] Wang X.M., Wen Y.Y., Liu X.C., Wen D., Long Y.X., Zhao P., Liu P.A., Zhong J. (2021). Protection Effect and Vacancy of the Ecological Protection Redline: A Case Study in Guangdong-Hong Kong-Macao Greater Bay Area, China. Remote Sens..

[4] Moran-Ordonez A., Hermoso V., Martinez-Salinas A. (2022). Multi-objective forest restoration planning in Costa Rica: Balancing landscape connectivity and ecosystem service provisioning with sustainable development. J. Environ. Manag..

[5] Costanza R., de Groot R., Farber S., Grasso M., Hannon B., Limburg K., Naeem S., Paruelo J., Raskin R., Sutton P. (1998). The value of the world’s ecosystem services and natural capital. Ecol. Econ..

[6] Reid W.V., Mooney H.A., Capistrano D., Carpenter S.R., Chopra K., Cropper A., Dasgupta P., Hassan R., Leemans R., May R.M. (2006). Nature: The many benefits of ecosystem services. Nature.

[7] Xie H.L., Zhang Y.W., Choi Y., Li F.Q. (2020). A Scientometrics Review on Land Ecosystem Service Research. Sustainability.

[8] Li Y.C., Abbas Z., Chen D.Y., Zhu Z.Y., Guo H.J., Zhao Y.L. (2022). The Eco-Environmental Changes in Typical Coastal Zones of Southern China From 1987 to 2020: A Case Study of Guangdong Coastal Counties. Front. Environ. Sci..

[9] Cao S.X., Liu Z.X., Li W.M., Xian J.L. (2021). Balancing ecological conservation with socioeconomic development. Ambio.

[10] Chen B.S., Zhao B., Li Y., Yu Q.Y., Zhao B.J., Tan J.Y., Wen C.H. (2022). Spatiotemporal Evolution and Factors Influencing Ecological Civilization Development in Chinese Watersheds. Int. J. Environ. Res. Public Health.

[11] Cao Y., Kong L., Zhang L., Ouyang Z. (2021). The balance between economic development and ecosystem service value in the process of land urbanization: A case study of China’s land urbanization from 2000 to 2015. Land Use Policy.

[12] Song F., Su F.L., Mi C.X., Sun D. (2021). Analysis of driving forces on wetland ecosystem services value change: A case in Northeast China. Sci. Total Environ..

[13] Gascoigne W.R., Hoag D., Koontz L., Tangen B.A., Shaffer T.L., Gleason R.A. (2011). Valuing ecosystem and economic services across land-use scenarios in the Prairie Pothole Region of the Dakotas, USA. Ecol. Econ..

[14] Polasky S., Segerson K. (2009). Integrating ecology and economics in the study of ecosystem services: Some lessons learned. Annu. Rev. Resour. Econ..

[15] Weiskopf S.R., Myers B.J.E., Arce-Plata M.I., Blanchard J.L., Ferrier S., Fulton E.A., Harfoot M., Isbell F., Johnson J.A., Mori A.S. (2022). A Conceptual Framework to Integrate Biodiversity, Ecosystem Function, and Ecosystem Service Models. Bioscience.

[16] Hamel P., Chaplin-Kramer R., Sim S., Mueller C. (2015). A new approach to modeling the sediment retention service (InVEST 3.0): Case study of the Cape Fear catchment, North Carolina, USA. Sci. Total Environ..

[17] Choudhary A., Deval K., Joshi P.K. (2021). Study of habitat quality assessment using geospatial techniques in Keoladeo National Park, India. Environ. Sci. Pollut. Res..

[18] Bao Y.B., Liu K., Hu T., Hu S. (2015). Effects of Land Use Change on Habitat Based on InVEST Model. Arid Zone Res..

[19] Wang M., Di Y., Wang J.D. (2022). Ecosystem services and trade-offs and synergy analysis in Tianjin under different land use scenarios. J. Beijing For. Univ..

[20] Guo Y., Li P., Cheng W.J., Li X.W. (2022). Evaluation and complex relations analysis of ecosystem services based on spatial-temporal change of land use in Dongting Lake. Acta Sci. Circumstantiae.

[21] Wei P.J., Wu M.H., Jia Y.L., Gao Y.Y. (2022). Spatiotemporal variation of water yield in the upstream regions of the Shule River Basin using the InVEST Model. Acta Ecol. Sin..

[22] Zhang T.J., Zhang S.P., Cao Q., Wang H.Y., Li Y.L. (2022). The spatiotemporal dynamics of ecosystem services bundles and the social-economic-ecological drivers in the Yellow River Delta region. Ecol. Indic..

[23] Yu Y.H., Sun X.Q., Wang J.L., Zhang J.P. (2022). Using InVEST to evaluate water yield services in Shangri-La, Northwestern Yunnan, China. Peerj.

[24] Song X., Liu Y., Zhu X., Cao G., Chen Y., Zhang Z., Wu D. (2022). The impacts of urban land expansion on ecosystem services in Wuhan, China. Environ. Sci. Pollut. Res..

[25] Adelisardou F., Jafari H.R., Malekmohammadi B., Minkina T., Zhao W., Karbassi A. (2021). Impacts of land use and land cover change on the interactions among multiple soil-dependent ecosystem services (case study: Jiroft plain, Iran). Environ. Geochem. Health.

[26] Mei Y., Kong X., Ke X., Yang B. (2017). The impact of cropland balance policy on ecosystem service of water purification—A case study of Wuhan, China. Water.

[27] Nel L., Boeni A.F., Prohaszka V.J., Szilagyi A., Kovacs E.T., Pasztor L., Centeri C. (2022). InVEST Soil Carbon Stock Modelling of Agricultural Landscapes as an Ecosystem Service Indicator. Sustainability.

[28] Ma T., Li X., Bai J., Ding S., Zhou F., Cui B. (2019). Four decades’ dynamics of coastal blue carbon storage driven by land use/land cover transformation under natural and anthropogenic processes in the Yellow River Delta, China. Sci. Total Environ..

[29] Deng C.X., Liu J.Y., Liu Y.J., Li Z.W., Nie X.D., Hu X.Q., Wang L.X., Zhang Y.T., Zhang G.Y., Zhu D.M. (2021). Spatiotemporal dislocation of urbanization and ecological construction increased the ecosystem service supply and demand imbalance. J. Environ. Manag..

[30] Chuai X., Huang X., Lai L., Wang W., Peng J., Zhao R. (2013). Land use structure optimization based on carbon storage in several regional terrestrial ecosystems across China. Environ. Sci. Policy.

[31] Zhang B., Li L., Xia Q.Y., Dong J. (2022). ”Three line” under the constraints of land use change and its effect on carbon—In wuhan city circle as an example. Acta Ecol. Sin..

[32] Ke X.L., Tang L.P. (2019). The impact of urban expansion and cultivated land protection coupling on carbon storage in terrestrial ecosystems: Taking Hubei Province as an example. Acta Ecol. Sin..

[33] Ma F.Z. (2021). Evaluation of Ecosystem Service in the Core Water Source Area of the Middle Route of the South-North Water Diversion Project Based on InVEST Model. Master’s Thesis.

[34] Zhou L.L. (2020). Assessment and Multi-Scenario Simulation of Wetland Ecosystem Services in the Upper Reaches of the Yangtze River. Ph.D. Thesis.

[35] Liu Y.N., Kong L.Q., Xiao Y., Zheng H. (2018). Effects of Landscape Pattern Changes on Ecosystem Water Purification Service in the Yangtze River Basin. Environ. Prot. Sci..

[36] Mengist W., Soromessa T., Feyisa G.L. (2021). Landscape change effects on habitat quality in a forest biosphere reserve: Implications for the conservation of native habitats. J. Clean. Prod..

[37] Huang L.Y., Liu S.H., LI J. (2019). Spatial and Temporal Dynamics of Urban Ecological Land Use and Its Related Driving Forces: A Case Study of Wuhan City. Resour. Environ. Yangtze Basin.

[38] Xia Y., Zhang Y.Y., Li E.H., Cai X.B. (2022). Spatio-temporal Evolution and Prediction of Habitat Quality in Four Lakes Basin of Jianghan Plain. Resour. Environ. Yangtze Basin.

[39] Lu S., Bai X., Li W., Wang N. (2019). Impacts of climate change on water resources and grain production. Technol. Forecast. Soc. Chang..

[40] Yang X., Chen R.S., Meadows M.E., Ji G.X., Xu J.H. (2020). Modelling water yield with the InVEST model in a data scarce region of northwest China. Water Supply.

[41] Wei P.J., Chen S.Y., Wu M.H., Deng Y.F., Xu H.J., Jia Y.L., Liu F. (2021). Using the InVEST Model to Assess the Impacts of Climate and Land Use Changes on Water Yield in the Upstream Regions of the Shule River Basin. Water.

[42] Jacobs S.R., Breuer L., Butterbach-Bahl K., Pelster D.E., Rufino M.C. (2017). Land use affects total dissolved nitrogen and nitrate concentrations in tropical montane streams in Kenya. Sci. Total Environ..

[43] Rasheed T., Bilal M., Nabeel F., Adeel M., Iqbal H.M.N. (2019). Environmentally-related contaminants of high concern: Potential sources and analytical modalities for detection, quantification, and treatment. Environ. Int..

[44] Zeng C., Liu Y., Stein A., Jiao L. (2015). Characterization and spatial modeling of urban sprawl in the Wuhan Metropolitan Area, China. Int. J. Appl. Earth Obs. Geoinf..

[45] Weber D., Schaepman-Strub G., Ecker K. (2018). Predicting habitat quality of protected dry grasslands using Landsat NDVI phenology. Ecol. Indic..

[46] Hao R., Yu D., Liu Y., Liu Y., Qiao J., Wang X., Du J. (2017). Impacts of changes in climate and landscape pattern on ecosystem services. Science of the Total Environ..

[47] Wu L.L. (2021). Regional economic transformation in central and western China from the perspective of ecological and environmental protection. J. Environ. Prot. Ecol..

[48] Tao Q., Gao G.H., Xi H.H., Wang F., Cheng X.B., Ou W.X., Tao Y. (2022). An integrated evaluation framework for multiscale ecological protection and restoration based on multi-scenario trade-offs of ecosystem services: Case study of Nanjing City, China. Ecol. Indic..

[49] Zhang H. (2019). Research on the construction of Zhaoqing urban ecological infrastructure based on ecological security. Appl. Ecol. Environ. Res..

